# A real-world implementation of asthma clinic: Make it easy for asthma with Easy Asthma Clinic

**DOI:** 10.1016/j.waojou.2022.100699

**Published:** 2022-10-07

**Authors:** Watchara Boonsawat, Kittisak Sawanyawisuth

**Affiliations:** Department of Medicine, Khon Kaen University, Khon Kaen, 40002, Thailand

**Keywords:** Multidisciplinary, Controlled, Inhaled corticosteroid, Pragmatic study, Asthma

## Abstract

**Background and objective:**

Asthma is a common disease. Although several practice guidelines for asthma exist, good control is still problematic, particularly in developing countries. The Easy Asthma Clinic (EAC) was established in 2004 with the aim of providing simplified asthma guidelines, a multidisciplinary approach, and an online database. This study aimed to evaluate the outcomes of EAC in a real-world setting.

**Method:**

Clinical data were collected from the EAC database between 2004 and 2017. Treatment data and asthma control data were evaluated during the study period.

**Results:**

In all, 358 182 patients with asthma were treated at EAC in 1171 hospitals throughout Thailand during the 14-year period. For 3 264 117 visits, inhaled corticosteroid (ICS) was given at the highest percentage (average of 50.00%) with an average percentage of controllers at 75.08% and a trend of increasing treatment (coefficient 0.007; p < 0.001). The percentage of controlled asthma also increased from 20.48% to 27.76% with a coefficient of 0.015 (p for trend <0.001).

**Conclusion:**

The EAC may facilitate controller use in patients with asthma thereby increasing asthma control according to a large sample size and long longitudinal study.

## Introduction

Asthma is a common disease with an incidence rate of 43.12 million cases reported worldwide in 2017.^1^ It is related to several conditions and may lead to emergency room visits or admission due to uncontrolled asthma.^2^, ^3^, ^4^, ^5^, ^6^, ^7^ While asthma mortality decreased from 1992 to 2017, incidence or disability-adjusted life years (DALYs) did not change significantly during the same period (r = −0.71; p = 0.114 and r = −0.80; p = 0.058).^1^ These figures may indicate that physicians need to improve asthma management and quality of care. To achieve this, international and local guidelines have been established. Several factors that may hinder the efficacy of asthma guidelines for good management include the complexity of the guidelines, patients’ perceptions of them, and insufficient time for physicians, particularly in resource-limited settings.^8^ A study from Ethiopia found that uncontrolled asthma was highly prevalent at 71.67%, yet only 38.5% of doctors followed the Global Strategy for Asthma Management and Prevention (GINA) guidelines, and even fewer, 14.5%, had asthma action plans with patients.^9^ A survey from Thailand found that only 8% of patients with asthma experienced good control according to the GINA guidelines, and inhaled corticosteroids (ICS) were used by only 6.7% of patients.^10^

**Fig. 1:**
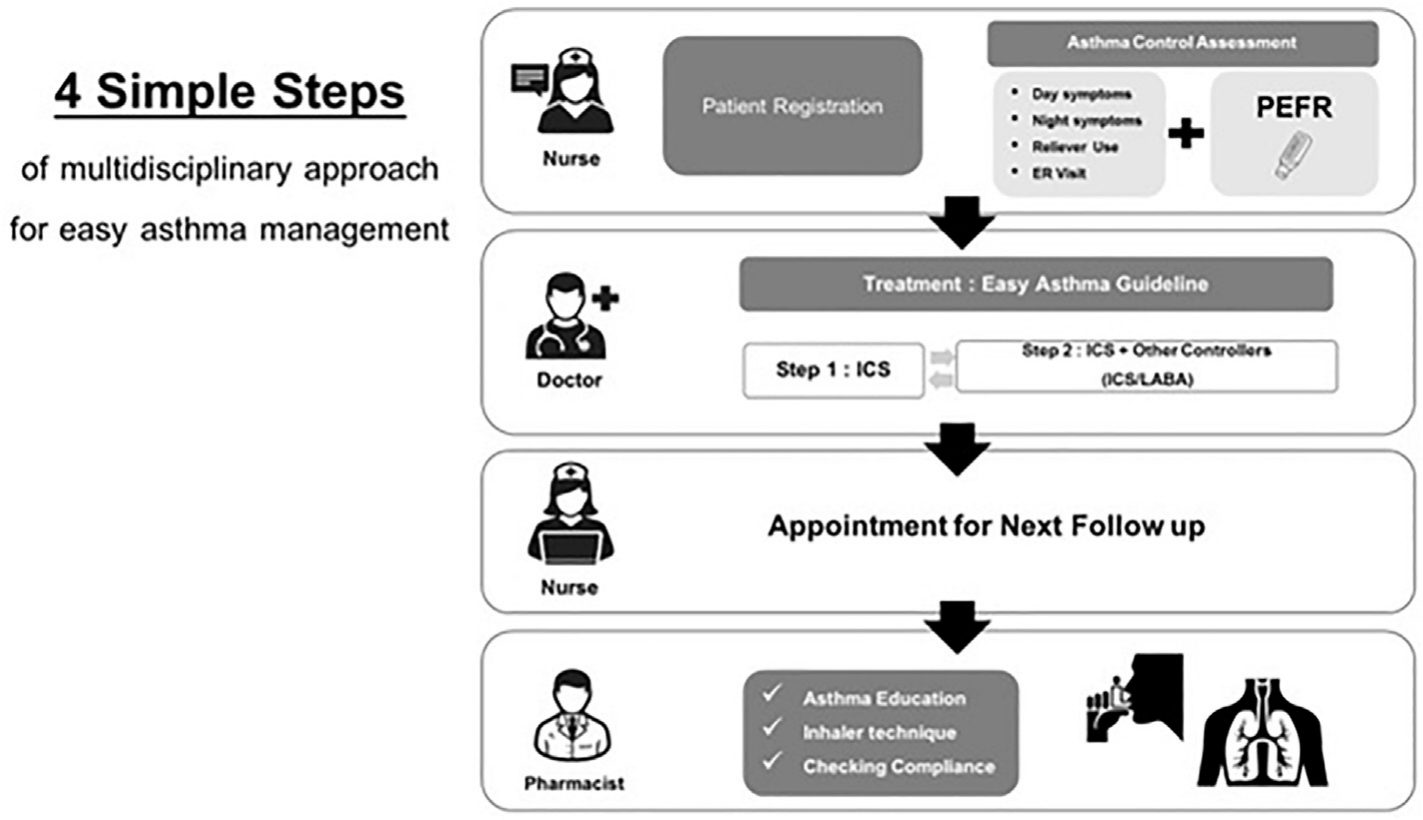
Shows the steps of the Easy Asthma Clinics.

To overcome these obstacles, the authors established Easy Asthma Clinic (EAC) in 2004. The aim of the EAC was to simplify asthma guidelines, implement a multidisciplinary approach, and create an online database (Fig. 1). To simplify guidelines, patients with asthma were assessed using 4 questions that addressed daytime symptoms, nocturnal symptoms, reliever use, and emergency room (ER) visits for the previous 4r weeks and peak-expiratory flow (PEF) measurement at the visit. The EAC has a multidisciplinary team including physicians (general practitioners or internists), nurses, and pharmacists. Four steps are included: 1) registration and asthma-control assessment by nurses; 2) evaluation and treatment by physicians; 3) appointments with nurses; and 4) asthma education and device education by clinically-trained pharmacists. Asthma education comprised 2 important messages: 1) that asthma is an inflammatory disease and 2) that asthma treatments included controller and reliever medications provided to the patient. The rationales for asthma treatment followed a previous study^11^ and involve 2 steps. In step 1, ICS 500 μg/d were administered, and in step 2, ICS 500 μg/d plus other controllers such as ICS/long-acting beta-2 agonist (ICS/LABA), antileukotriene, or theophylline were used. These 2 steps are bidirectional. Stepping up is indicated if symptoms are uncontrolled or partially controlled as demonstrated by the presence of any item of the above questions or PEF less than 80% of expected values. To step down, symptoms and PEF should be controlled for at least one year. The online database monitored individual patient and overall asthma control in each hospital. This study aimed to evaluate and report the outcomes of the EAC.

## Methods

This study was a real-world study conducted by retrieving clinical data from the EAC database (http://eac2.dbregistry.com). The inclusion criteria were patients diagnosed with asthma who had been treated at an EAC anywhere Thailand. Those patients with incomplete data were excluded. The study period was between 2004 and 2017.

Diagnosis of asthma was made according to the GINA guidelines.^12^ Eligible patients were evaluated for treatment and asthma control at each visit. Treatment outcomes were classified as controlled, partially controlled, and uncontrolled. Definitions of asthma control were classified by the GINA guidelines^12^ as follows: controlled comprised less than twice a week for daytime symptoms and reliever use less than twice a week, no limitations of activities, no nocturnal symptoms, normal PEF or FEV1, and no exacerbations; partially controlled was 1 or 2 unfavourable features of the controlled criteria; and uncontrolled was defined by the presence of 3 or more unfavourable features of the controlled criteria.^12^

Data were reported as numbers and percentages of controller treatment including ICS, ICS/LABA, and overall controller percentage and treatment outcomes as controlled, partially controlled, and uncontrolled. Additionally, data for treatments and treatment outcomes were reported by year. A P value for trend was calculated for each treatment and asthma control by using a trend analysis for proportions. Coefficients or beta and p values were reported. All statistical analyses were performed using STATA software (College Station, Texas, USA).

## Results

**Fig. 2:**
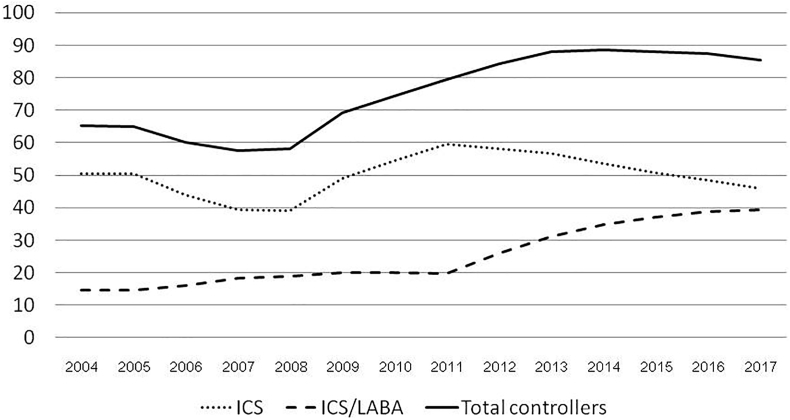
Shows the percentages of treatment with inhaled corticosteroids (ICS), treatment with inhaled corticosteroids/long-acting beta-2 agonists (ICS/LABA), and total percentage of controller prescriptions at the Easy Asthma Clinics by year.

**Fig. 3:**
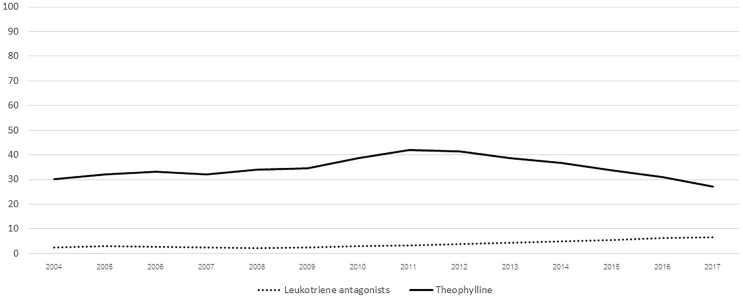
Shows the percentages of treatment with leukotriene antagonists and theophylline at the Easy Asthma Clinics by year.

There were 358 182 patients with asthma treated at the EAC in 1171 hospitals throughout Thailand during the 14-year period (Table 1). Of 3,264,117 visits, ICS was given at the highest percentage (average of 50.00%) and followed by ICS/LABA (24.95%) as shown in Table 2 and Fig. 2. The average percentage of controller prescription was 75.08% with a trend of increasing treatment of ICS/LABA and overall controller percentages (Fig. 2). Coefficients for ICS, ICS/LABA, and overall controller percentages were −0.003, 0.027, and 0.007 with a p value of <0.001 for these three treatments (Table 2). For other controllers, leukotriene antagonists were slightly increased from 2.43% in 2004 to 6.44% in 2017, while theophylline had a steady percentage (Fig. 3).

**Table 1:** Shows the numbers of hospitals, registered patients, and total patients treated at Easy Asthma Clinics by year

year	Hospitals	Registered patients	Total patients
2004	367	4144	4144
2005	413	7079	11,223
2006	443	7333	18,556
2007	501	8042	26,598
2008	561	14,884	41,482
2009	618	17,710	59,192
2010	896	40,812	100,004
2011	1062	60,565	160,569
2012	1101	45,815	206,384
2013	1130	40,225	246,609
2014	1145	32,425	279,034
2015	1151	29,329	308,363
2016	1160	27,074	335,437
2017	1171	22,745	358,182

**Table 2:** Showed numbers of visits, treatment with inhaled corticosteroid (ICS), treatment with inhaled corticosteroid/long acting beta 2 agonist (ICS/LABA), and total percentage of total controller prescription at the Easy Asthma Clinics by year

Year	Visits	ICS, n	%	ICS/LABA, n	%	% total controller
2004	9974	5038	50.51	1463	14.67	65.18
2005	24,570	12,382	50.39	3593	14.62	65.11
2006	38,472	16,915	43.97	6179	16.06	60.10
2007	46,648	18,392	39.43	8506	18.23	57.72
2008	72,282	28,288	39.14	13,611	18.83	58.07
2009	104,338	51,192	49.06	20,847	19.98	69.14
2010	202,263	109,906	54.34	40,368	19.96	74.39
2011	356,403	212,466	59.61	70,262	19.71	79.41
2012	424,394	246,897	58.18	110,918	26.14	84.44
2013	460,498	260,837	56.64	143,800	31.23	87.99
2014	472,066	253,623	53.73	164,455	34.84	88.71
2015	402,674	204,198	50.71	149,039	37.01	87.92
2016	354,376	171,690	48.45	137,256	38.73	87.43
2017	295,159	135,414	45.88	115,974	39.29	85.48
Total	3,264,117	1,727,238	50.00	986,271	24.95	75.08
Coefficient/P for trend (B/P)		B −0.003	<0.001	B 0.027	<0.001	B 0.007/P < 0.001

**Fig. 4:**
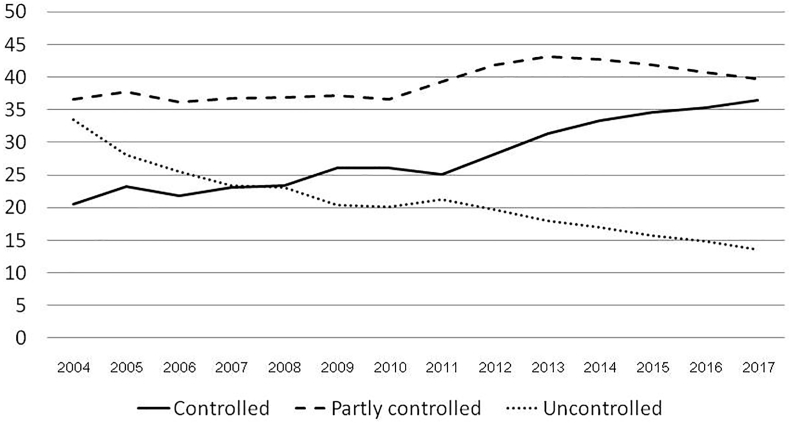
Showed percentages of patients with controlled, partially controlled, and uncontrolled in the Easy Asthma Clinics by year.

Regarding treatment outcomes, average percentages of controlled, partially controlled, and uncontrolled were 27.76%, 39.09%, and 21.00%, respectively (Table 3). Of 3 264 025 visits, percentage of controlled patients increased from 20.48% in 2004 to 36.47% in 2017 (Table 3 and Fig. 4), while percentages of partially controlled cases were increasing slightly from 36.57% to 39.72%. In contrast, the uncontrolled percentage was dramatically decreasing from 33.42% to 13.54% (Table 3 and Fig. 3). Coefficients for controlled asthma and partially controlled asthma were positive at 0.015 and 0.004 (p < 0.001), while the coefficient for uncontrolled asthma was negative at −0.011 (p < 0.001), as shown in Table 3.

**Table 3:** Shows the numbers of visits, numbers and percentages of patients with controlled, partially controlled, and uncontrolled cases treated at the Easy Asthma Clinics by year.

Year	Visits	Controlled, n	%	Partly controlled, n	%	Uncontrolled, n	%
2004	9974	2043	20.48	3647	36.57	3333	33.42
2005	24,570	5718	23.27	9263	37.70	6882	28.01
2006	38,466	8392	21.82	13,897	36.13	9831	25.56
2007	46,642	10,745	23.04	17,163	36.80	10,882	23.33
2008	72,282	16,875	23.35	26,657	36.88	16,674	23.07
2009	104,336	27,257	26.12	38,792	37.18	21,210	20.33
2010	202,250	52,806	26.11	74,110	36.64	40,732	20.14
2011	356,392	89,537	25.12	139,847	39.24	75,974	21.32
2012	424,391	119,604	28.18	177,737	41.88	83,676	19.72
2013	460,492	144,372	31.35	198,671	43.14	82,760	17.97
2014	472,055	157,187	33.30	201,569	42.70	80,132	16.98
2015	402,667	139,287	34.59	168,544	41.86	63,519	15.77
2016	354,367	125,392	35.38	144,455	40.76	52,640	14.85
2017	295,141	107,625	36.47	117,244	39.72	39,975	13.54
Total	3,264,025	1,006,840	27.76	1,331,596	39.09	588,220	21.00
Coefficient/P for trend (B/P)		B 0.015	<0.001	B 0.004	<0.001	B −0.011	<0.001

Note: There are missing data due to incomplete data collection; the total number of visits was not equal to the sum of the controlled, partially controlled, and uncontrolled cases column

## Discussion

The results of this study showed that EAC was able to facilitate controller use and improve asthma control with easy treatment strategies, a multidisciplinary approach, and an available online database.

As previously reported, one factor associated with uncontrolled asthma is using only a bronchodilator without a controller or ICS.^8^ A study in Ethiopia or Zemedkun et al found that only 3.8% of patients received ICS with or without LABA.^13^ ICS is recommended in all stages of asthma according to recent guidelines and several studies because asthma is a disease caused by inflammation of the airway.^14^, ^15^, ^16^ The EAC uses ICS as the first line treatment immediately after the diagnosis of asthma. Without the ICS, the adjusted odds ratio for uncontrolled asthma was 13.642 (95% confidence interval of 4.403, 42.22).^17^ This study found an overall increase in asthma control which may be explained by the increased use of ICS/LABA but not ICS alone as the coefficients of both controlled asthma and ICS/LABA were positive (0.015 and 0.027), while the coefficient of ICS alone was negative (−0.003) as shown in Table 2, Table 3 Additionally, these treatments may indicate more-severe asthma in the population.^8^ Importantly, treatment at EAC facilitates controller treatment or treatment including ICS in three-fourths of patients, which is much higher than controller treatment in the survey study (75.08% vs. 6.7%).^10^ Recently, the 2020 GINA guidelines recommended controller-based treatment for patients with asthma which the EAC project has used for 14 years.^18^ Note that other controllers may not be related to controlled rates as they had small proportions with slightly increased for only leukotriene antagonists (Fig. 3).

This study found that the percentage of controlled asthma cases was high with an increasing trend from 20.48% to 36.47%. Compared with previous studies, the average controlled percentage at the end of this study was slightly higher than previous reports from Ethiopia as a result of EAC strategies (from 24.2% to 29.6%).^8^^,^^19^^,^^20^ Reasons for these results included the use of ICS, PEF evaluation, and a multidisciplinary approach, particularly, education regarding the technique for using the device.^8^^,^^19^ This education is critical to improving ICS delivery to the airways. A previous study found that incorrect inhaler-device use was identified in 70.4% of cases.^20^ These results imply that asthma control may be improved by up to 70.4% if patients understand and use correct device techniques. Additionally, poor inhaler technique was significantly associated with lack of education in the device as well as poor asthma control with adjusted odds ratios of 4.96 (1.08–22.89) and 3.67 (1.85–7.23) and p values of 0.04 and 0.001 respectively.^20^ The EAC offered health education on the device and how to use it properly. Early use of ICSs and their fast step-up may be additional benefits of EAC. A meta-analysis found that simplified regimens improved patient compliance by 4% points (95% CI of 1.88, 6.16).^21^ These factors may improve asthma control in patients treated with the EAC concepts. Note that the proportion of partially controlled asthma initially increased up to 2013 and then progressively decreased (Table 3); these findings may be due to higher percentages of ICS/LABA (Table 2) resulting in higher controlled percentages but decreasing percentages of partially controlled asthma. These data suggest that ICS/LABA may be associated with well-controlled asthma.

There are several strengths of this study. First, this was a real-world, longitudinal 14-year study comprising a large cohort and covering more than 1000 hospitals throughout Thailand. Second, 358,182 patients with asthma participated at the EAC with over 3 000 000 visits. Third, the 4 steps used by the EAC provided more time for physicians to manage patients and also to see more patients. As there are nurses and pharmacists to assist with treatment, providing education, evaluation, and device training, physicians spend less time per patient. Therefore, physicians have more time to see more patients. Physicians at the EAC are general practitioners or internists. These data showed that patients’ asthma could be controlled by general practitioners or internists. Patients with more complex cases were referred to pulmonologists at higher level hospitals as per the national referral guidelines. Finally, this study simplified asthma guidelines for individual treatment with a multidisciplinary approach. However, several limitations must be noted. First, no control group for comparison was used in this study. Second, ICSs or other medications used in this study were not specific to any particular drugs. Finally, some factors, such as obstructive sleep apnoea, occupational asthma, or personal factors, were not evaluated.^22^, ^23^, ^24^, ^25^, ^26^, ^27^, ^28^

## Conclusion

The EAC may facilitate controller use in patients with asthma and result in an increasing trend of asthma control according to a large sample size and long longitudinal study.

## Abbreviations

DALYs, Disability-adjusted life years; EAC, Easy asthma clinic; FEV1, Forced expiratory volume during the first second; GINA, Global Strategy for Asthma Management and Prevention; ICS, Inhaled corticosteroid; LABA: Long acting beta 2 agonist; PEF, Peak expiratory.

## Funding

Not applicable.

## Availability of data and materials

Data are available as printed material and as electronic files in the hospital computer. Patients’ data protection.

## Authors' contribution

The authors are involved in the collection of the data and the writing of the manuscript. The authors read and approved the final manuscript.

## Ethics approval

The Institutional Review Board (IRB) of Khon Kaen University exempted the study from IRB ethics approval and informed consent as it used a de-identified data set of the electronic medical record system of the EAC system stripped of all Health Insurance Portability and Accountability Act (HIPPAA) identifiers.

## Consent to participate

Not applicable.

## Authors’ consent for publication

The authors provided consent for publication.

## Declaration of competing interest

The authors declare that the research was conducted in the absence of any commercial or financial relationships that could be construed as a potential conflict of interest.

## Acknowledgements

Not applicable.
